# Investigating the associations between intimate partner violence and nutritional status of women in Zimbabwe

**DOI:** 10.1371/journal.pone.0272038

**Published:** 2022-07-25

**Authors:** Jeanette Iman’ishimwe Mukamana, Pamela Machakanja, Hajo Zeeb, Sanni Yaya, Nicholas Kofi Adjei

**Affiliations:** 1 Institute of Peace, Leadership and Governance, Africa University, Mutare, Zimbabwe; 2 Leibniz Institute for Prevention Research and Epidemiology – BIPS, Bremen, Germany; 3 Health Sciences Bremen, University of Bremen, Bremen, Germany; 4 School of International Development and Global Studies, Faculty of Social Sciences, University of Ottawa, Ottawa, Canada; 5 Department of Public Health, Policy and Systems, University of Liverpool, Liverpool, United Kingdom; PLOS, UNITED KINGDOM

## Abstract

**Background:**

Intimate partner violence (IPV) against women and poor nutritional status are growing health problems in low and middle-income countries (LMICs). Moreover, violence against women has been shown to be associated with poor nutrition. This study investigated the relationship between IPV and nutritional status (i.e., underweight, overweight, and obesity) among women of reproductive age (15–49 years) in Zimbabwe.

**Methods:**

Pooled data from the 2005/2006, 2010/2011, and 2015 Zimbabwe Demographic Health Surveys (ZDHS) on 13,008 married/cohabiting women were analysed. Multinomial logistic regression models were used to examine the associations between the various forms of IPV and the nutritional status of women. We further estimated the prevalence of BMI ≥ 25.0 kg/m^2^ (overweight and obesity) by intimate partner violence type.

**Results:**

The mean BMI of women was 24.3 kg/m^2^, more than one-fifth (24%) were overweight and about 12% were obese. Forty-three percent (43%) of women reported to have ever experienced at least one form of intimate partner violence. More than one-third (35%) of women who reported to have ever experienced at least one form of intimate partner violence had a BMI ≥ 25.0 kg/m^2^ (p< 0.01). Relative to normal weight, women who had ever experienced at least one form of IPV (i.e., physical, emotional, or sexual) were more likely to be obese (aOR = 2.59; 95% CI = 1.05–6.39). Women’s exposure to any form of intimate partner violence was not significantly associated with the likelihood of being underweight or overweight relative to normal weight.

**Conclusions:**

The study findings show that women of reproductive age in Zimbabwe are at high risk of both IPV and excess weight. Moreover, we found a positive relationship between exposure to at least one form IPV and obesity. Public health interventions that target the well-being, empowerment and development of women are needed to address the complex issue of IPV and adverse health outcomes, including obesity.

## Introduction

Intimate partner violence (IPV) is a form of gender-based violence [1], mostly perpetrated against women [2,3]. This behaviour is assaultive and coercive [4,5], and it comes in the form of emotional, sexual, or physical abuse [6–9]. The various forms of abuse may co-exist [10]; for instance, physical abuse or violence is mostly accompanied by sexual violence, and the latter may also come along with emotional violence [10,11]. IPV is increasingly recognized as a relevant social and health problem by relevant institutions and organizations worldwide [12–14], due to its adverse impacts on victims [10,15], and society as a whole [7,16–19].

The prevalence of IPV is high in developing countries [1]. However, there is evidence of cross-country variations [20,21], where Zimbabwe has been found to be one of the countries in sub-Saharan Africa with the highest prevalence of IPV [21,22]. It is estimated that approximately 35% of women had experienced physical violence from the age of 15 and 14% had experienced sexual violence [23]. In a recent study in Zimbabwe, Mukamana and colleagues found a substantial rise in the prevalence of IPV from 40.9% in 2010 to 43.1% in 2015 [1].

Violence against women as a health problem [16,17] has been shown to be one of the leading causes of both medical diagnosed and non-medical explainable physical, mental, and gynecological health problems [7,24–27]. Also, it remains a symptom of gendered power relations [28,29], which may be a predictor of women’s health [30,31], including stressful conditions [28,32], and nutritional status such as underweight, overweight, and obesity [18].

The issue of obesity is becoming a worldwide problem [33], increasingly also in developing countries [34]. Globally, overweight and obesity among female adults have increased from 29.8% to 38.0% between 1980 and 2013 respectively [32]. In Sub-Saharan Africa, the prevalence of overweight and obesity has been rising at an alarming rate [35], and women are the most affected [35]. In Zimbabwe, for instance, a recent study showed an increase in the prevalence of overweight and obesity from 25.0% in 2005 to 36.6% in 2015 [36]. The authors also observed socioeconomic and demographic differences in overweight and obesity among women of reproductive age. Differences in experiencing obesity and overweight among socioeconomic subgroups [37] may be linked to IPV in complex ways. For example, prior evidence suggests that abused women may end up suffering from depression [38], and may hence seek consolation in overeating [39]. In rich food environments, they tend to consume energy-dense foods [40], which has been shown to be a risk factor for obesity [18,40]. Furthermore, there is evidence that physical and sexual violence against women may predict excessive weight gain and poor nutrition [41,42], where some abused women tend to suffer from depression, increased anxiety, loss of appetite, and eating disorders with limited caloric intake [43,44]. The stress suffered by abused women has been shown to increase oxidative stress and metabolic syndrome including obesity and cardiovascular disease [44,45], which are also risk factors for anemia and underweight [30,38]. IPV thus contributes to the risk of poor nutrition outcomes, especially where abusive male partners withhold food as a form of punishment to their female partners [46].

From the above discussions, it is clear from the literature that there is a relationship between IPV and women’s health [47,48]. While some studies have examined the relationship between dietary knowledge, the attitude of behaviours, socio-demographic factors, and IPV [18,31,49,50], no study has investigated the association between IPV and the nutritional status of women in Zimbabwe. This study, therefore, sought to explore the relationship between IPV and nutritional status (i.e., underweight, overweight, and obesity) among women of reproductive age in Zimbabwe.

## Materials and methods

### Data

The analysis was based on pooled data of married/cohabiting women from the 2005/2006, 2010/2011, and 2015 Zimbabwe Demographic Health Surveys. The surveys were conducted by the Zimbabwe National Statistical Agency in collaboration with other international organizations, and they were nationally representative surveys of men and women in their reproductive age. The surveys employed a two-stage stratified cluster sampling technique based on census enumeration areas (EAs) and household samples in both rural and urban areas. The first stage was the selection of EAs with probability proportional to the size and the second stage involved household sampling. The analysis was limited to non-pregnant women of reproductive age with valid weight and height measurements. Pregnant women were excluded to avoid a misleading picture of the issue of overweight and obesity during pregnancy [36]. The samples after the exclusion were (survey year: 2005/2006; n = 4,031), (survey year: 2010/2011; n = 4,211) and (survey year: 2015; n = 4,766), with a pooled total (N = 13,008) for the final analysis.

### Measurement of the outcome variable

The outcome variable for this study was the nutritional status of women (i.e., underweight, normal weight, overweight, and obesity). The body mass index (BMI; weight (kg)/height (m) squared) was used to assess the nutritional status of women [51], and it is commonly used to classify underweight, overweight, and obesity in adults [52,53]. Respondents were classified according to the BMI criteria of the World Health Organisation (WHO): a) underweight, BMI < 18.5 kg/m^2^; b) normal weight, BMI of 18.5–24.9 kg/m^2^; c) overweight, BMI of 25.0–29.9 kg/m^2^ and d) obesity, BMI ≥ 30.0 kg/m^2^ [54]. In the surveys, participants’ standing heights were measured using a measuring board and their weights were taken using the United Nations Children’s Fund (UNICEF) electronic scale with a digital display.

### Independent variable

The independent variable in this study was IPV. The measurement of IPV in the surveys was based on the modified Conflict Tactics (CTS2) [23,55,56] and was administered following standard guidelines for research on domestic violence set by the World Health Organisation [57]. The questions posed to women measure included “did your husband/partner ever: slap, push, shake, punch, beat, kick or try to strangle you, throw something at you, threaten you using a harmful object?” These questions were used to derive physical violence. Sexual violence was assessed by the questions “did your husband/partner ever: physically force you to have sexual intercourse even when you did not want? Or force you with threats to perform any sexual acts you did not want?” Psychological violence was assessed using questions such as “did your husband/partner humiliate you in front of others, threaten to hurt you or those close to you with harm?” Responses were categorized as physical, emotional, sexual, physical or emotional, physical or sexual, emotional or sexual, and physical, sexual or emotional. Answers in the affirmative were coded as “1”, while women who never experienced any of the aforementioned forms of IPV were coded as “0”.

### Covariates

**Fig 1:**
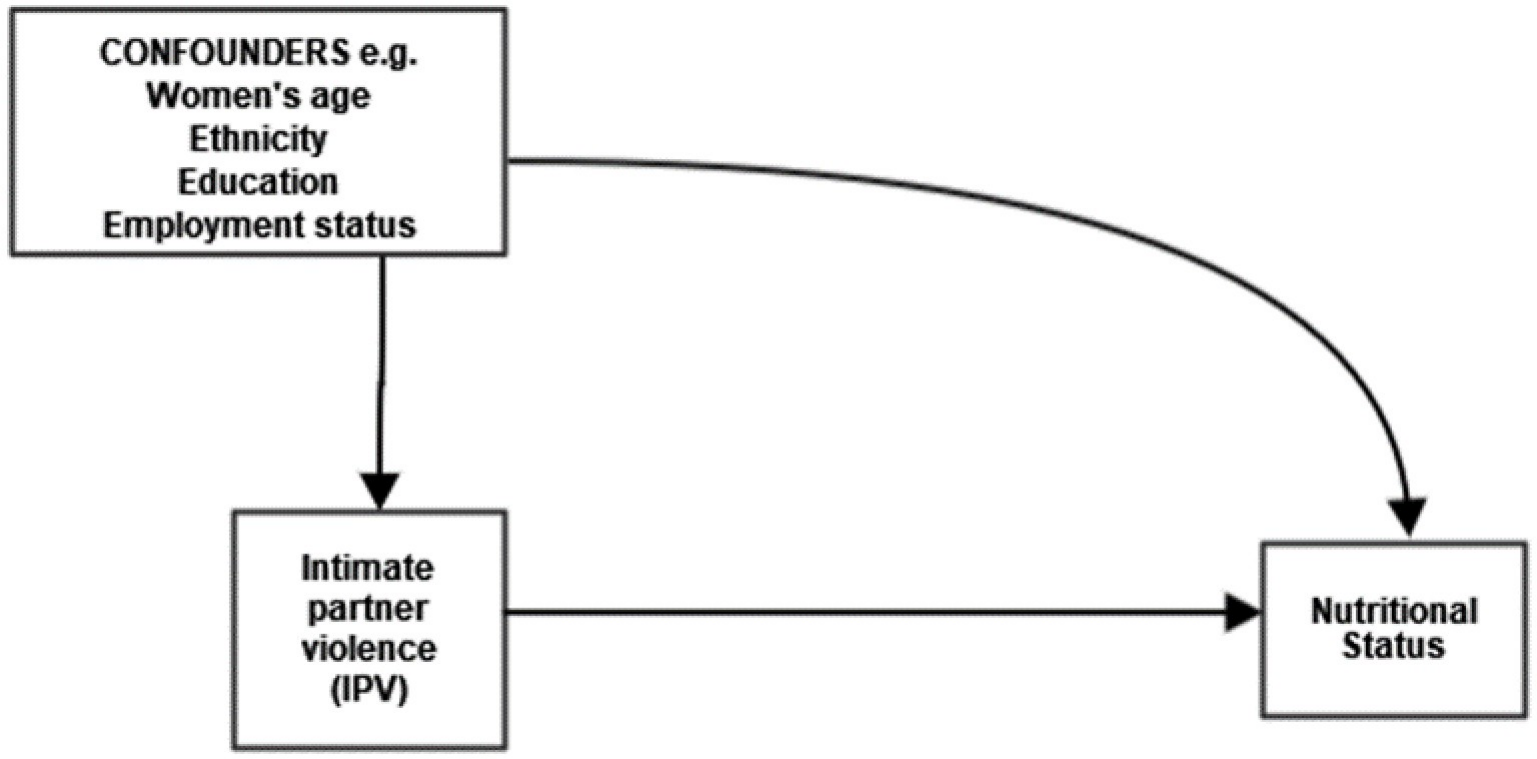
Directed acyclic graph for the current study.

In the adjusted regression models, we controlled for the following socio-demographic and economic variables: age (15–29, 20–24, 25–29, 30–34, 35–39, 40+); marital status (married, cohabiting); place of residency (rural, urban), educational level (no education, primary, secondary and higher); parity (<2, 2–3, 4–5, 6+); employment status (not currently employed, currently employed); and wealth index (poorest, poorer, middle, richer), guided by a directed acyclic graph (Fig 1). The wealth index in the DHS is usually computed using durable goods, household characteristics and basic services. All the variables were obtained from either the individual women’s or the household questionnaires.

### Statistical analysis

First, basic descriptive statistics were performed to obtain the mean, frequency, and percentages of the dependent, independent, and some control variables. Second, percentages (%) were used to describe the prevalence of BMI ≥ 25.0 kg/m^2^ (overweight and obesity) and the various forms of IPV. Differences in prevalence were examined using chi-square test. Third, we estimated the prevalence of IPV among women who experienced at least one type of abuse (i.e. physical, sexual or emotional) by nutritional status (i.e., underweight, normal weight, overweight and obese). In the second part of the analysis, multinomial logistic regression models were used to examine the associations between the various forms of IPV and the nutritional status of women. The prevalence and adjusted odd ratios (aOR) with 95% confidence intervals (95% CI) was calculated using Stata Version 14 (Stata Corp, College Station, Texas, USA). The dataset was weighted to account for differences in the sampling design.

## Results

### Distribution of selected characteristics

The distribution of respondents’ characteristics is shown in Table 1. Overall, the mean age of women was approximately 30 years. Most women (64%) reported having secondary or higher education. On average, women had three live births, and about 67% lived in rural areas. Regarding economic status, more than half (61%) were not in paid employment, and 41% reported middle economic class.

**Table 1:** Percentage distribution of the characteristics of women (15–49 years) in Zimbabwe, pooled data, 2005–2015 (*n* = 13,008).

Variables	N = 13008	% (95% CI)	Mean	(SD)	Min	Max
**Anthropometry**						
BMI (Kg/m2)			24.31	(4.64)	13.27	57.74
Underweight, or BMI<18.5	665	5.11 (4.73–5.50)				
Normal weight, or BMI 18.5≤BMI<25	7733	59.45 (58.59–60.29)				
Over weight, or 25≤BMI<30	3068	23.59 (22.85–24.32)				
Obese, or BMI≥30	1542	11.85 (11.3–12.4)				
**Intimate Partner Violence, by type**						
Physical						
Ever	3666	28.18 (27.41–28.96)				
Never	9342	71.82 (71.03–72.58)				
Emotional						
Ever	3650	28.06 (27.28–28.84)				
Never	9358	71.94 (71.11–72.71)				
Sexual						
Ever	1639	12.60 (12.03–13.18)				
Never	11369	87.40 (86.81–87.96)				
Physical or Emotional						
Ever	5219	40.12 (39.2–40.96)				
Never	7789	59.88 (59.03–60.72)				
Physical and Emotional						
Ever	2097	16.12 (15.49–16.76)				
Never	10911	83.88 (83.22–84.50)				
Physical or Sexual						
Ever	4330	33.29 (32.47–34.10)				
Never	8678	66.71 (65.89–67.52)				
Physical and Sexual						
Ever	957	7.50 (7.04–7.96)				
Never	12033	92.50 (92.03–92.95)				
Emotional or Sexual						
Ever	4296	33.03 (32.21–33.84)				
Never	8714	66.97 (66.15–67.78)				
Emotional and Sexual						
Ever	993	7.63 (7.18–8.10)				
Never	12015	92.37 (91.89–92.81)				
Physical or Emotional or Sexual						
Ever	5615	43.17 (42.31–44.02)				
Never	7393	56.83 (55.97–57.68)				
All three						
Ever	725	5.57 (5.18–5.98)				
Never	12283	94.43 (94.01–94.81)				
**Sociodemographic controls**						
Age			30.36	(7.96)	15.0	49.0
15–19	824	6.33 (5.92–6.76)				
20–24	2696	20.73 (20.03–21.43)				
25–29	3038	23.35 (22.62–24.09)				
30–34	2639	20.29 (19.59–20.98)				
35–39	1776	13.65 (13.06–14.25)				
40+	2035	15.64 (15.02–16.28)				
Marital Status						
Married	12442	95.65 (95.28–95.99)				
Cohabiting	566	4.35 (4.01–4.71)				
Parity			2.80	(1.87)	0.0	13.0
<2	3341	25.68 (24.93–26.44)				
2–3	5880	45.20 (44.34–46.06)				
4–5	2670	20.53 (19.83–21.23)				
6+	1117	8.59 (8.11–9.08)				
Place of residence						
Urban	4340	33.36 (32.55–34.18)				
Rural	8668	66.64 (65.81–67.44)				
Educational Level						
No education	363	2.79 (2.51–3.08)				
Primary	4330	33.29 (32.47–34.10)				
Secondary and higher	8315	63.92 (63.09–64.74)				
Employment Status						
Not currently employed	7950	61.12 (60.27–61.95)				
Currently employed	5058	38.88 (38.04–39.72)				
Wealth (Index)						
Poorest	2701	20.76 (20.01–21.47)				
Poorer	2482	19.08 (18.40–19.76)				
Middle	5394	41.47 (40.61–42.31)				
Richer	2431	18.69 (18.02–19.36)				

The mean BMI of women was 24.3 kg/m^2^ (Table 1). A high proportion of women had normal weight (59%), more than one-fifth were overweight (24%) and about 12% were obese. The results further showed that more than one-third (43%) of women reported to have ever experienced at least one form of intimate partner violence, and large proportions ever experienced physical (28%), emotional (28%), and sexual (13%) violence. More than one-third reported any physical or emotional violence (40%) and any emotional or sexual violence (33%).

**Fig 2:**
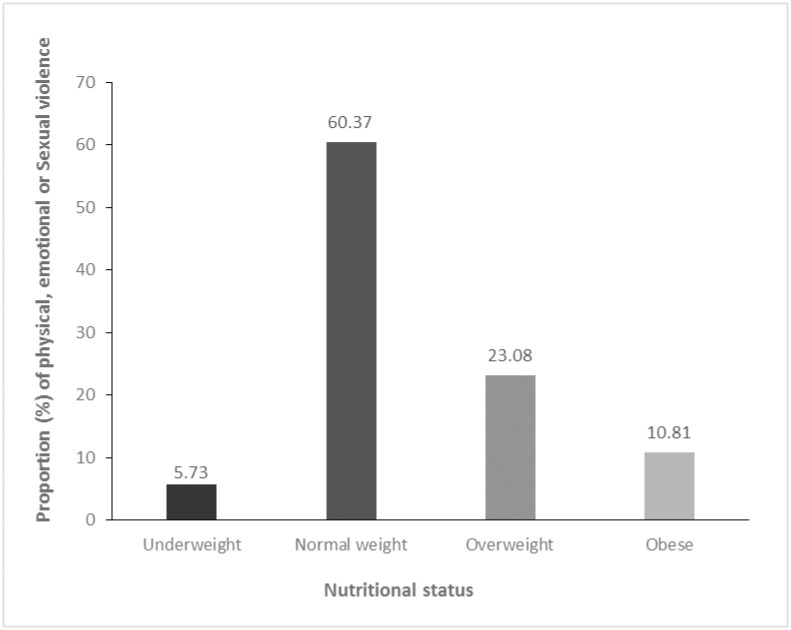
Proportion (%) of physical, emotional or sexual violence against women of reproductive age (15–49 years) by nutritional status, Zimbabwe, pooled data, 2005–2015.

**Fig 3:**
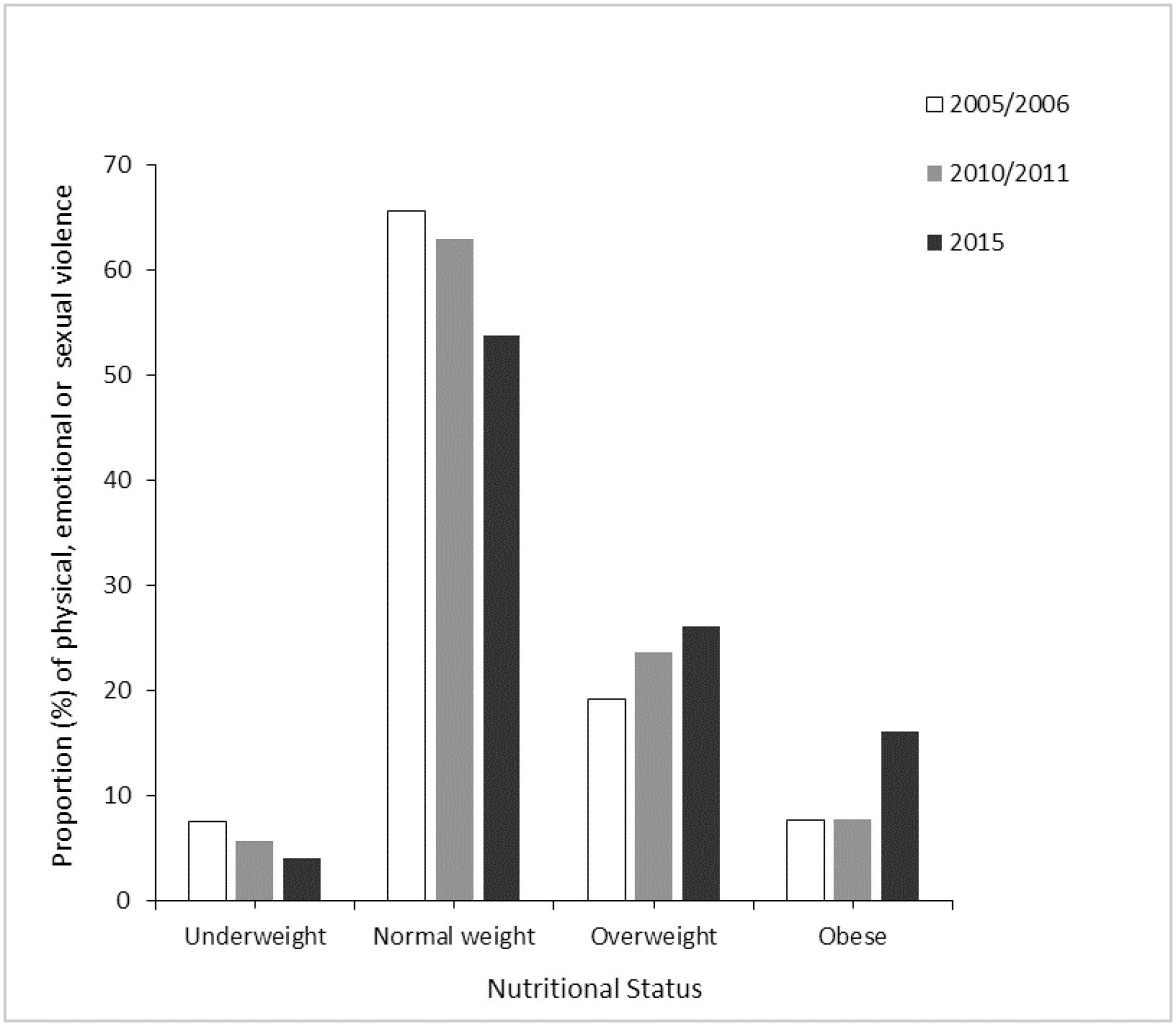
Proportion (%) of physical, emotional or sexual violence against women of reproductive age (15–49 years) by nutritional status and survey year, Zimbabwe.

In Table 2, the results of the prevalence of BMI ≥ 25.0 kg/m^2^ (overweight and obesity) by intimate partner violence type are shown. In general, more than one-third (35%) of women who reported to have ever experienced at least one form of intimate partner violence (i.e., physical emotional, or sexual) had a BMI ≥ 25.0 kg/m^2^ (p< 0.01). Similarly, more than one-third of women who ever experienced sexual (33%), any physical or emotional (34%), and any physical or sexual (33%) violence reported being overweight or obese. The overall proportion (%) of any form of intimate partner violence (i.e., physical, sexual, or emotional) was generally high (60%) among women who had normal weight (Fig 2). Meanwhile, the trend analysis by survey year showed a decline from 65.6% in 2005/2006 to 53.7% (Fig 3).

**Table 2:** Prevalence of BMI ≥ 25.0 kg/m^2^ (overweight and obesity) among women of reproductive age (15–49 years) by intimate partner violence type, Zimbabwe, pooled data, 2005–2015.

Variables	BMI≥25 Kg/m2 (%)	*P* value*
**Intimate Partner Violence, by type**		
Physical		< 0.001
Ever	31.86 (30.37–33.38)	
Never	36.84 (35.87–37.82)	
Emotional		0.244
Ever	34.66 (33.12–36.21)	
Never	35.75 (34.77–36.72)	
Sexual		< 0.05
Ever	33.07 (30.83–35.38)	
Never	35.78 (34.90–36.66)	
Physical or Emotional		< 0.001
Ever	33.89 (32.62–35.19)	
Never	36.62 (35.41–37.55)	
Physical and Emotional		< 0.001
Ever	31.66 (29.70–33.68)	
Never	36.16 (35.26–37.07)	
Physical or Sexual		< 0.001
Ever	32.49 (31.11–33.90)	
Never	36.91 (35.89–37.93)	
Physical and Sexual		< 0.05
Ever	31.17 (28.24–34.05)	
Never	35.79 (34.94–36.65)	
Emotional or Sexual		< 0.05
Ever	34.08 (32.67–35.50)	
Never	36.11 (35.10–37.12)	
Emotional and Sexual		0.538
Ever	34.54 (31.64–37.55)	
Never	35.51 (34.66–36.37)	
Physical or Emotional or Sexual		< 0.01
Ever	35.44 (32.66–37.10)	
Never	36.62 (35.52–37.72)	
All three		0.130
Ever	32.82 (29.50–36.33)	
Never	35.55 (34.75–36.44)	

Note -

* p values are based on the χ2 test, data are % (95% CI—Clopper-Pearson).

### Multinomial logistic regression

The adjusted odd ratios (aOR) and 95% confidence intervals for the associations between intimate partner violence and the nutritional status of women are shown in Table 3. The multinomial regression model estimated the relative risk ratios of the relationships between intimate partner violence and body mass index (BMI) comparing underweight, overweight, and obesity to normal weight. In the model, we adjusted for socioeconomic factors (categorical, as shown in Table 1) and other behavioural risk factors including smoking status (yes or no), alcohol consumption (yes or no), and media exposure (yes or no).

**Table 3:** Multinomial logistic regression of the association between intimate partner violence and nutritional status of women (15–49 years), Zimbabwe, pooled data, 2005–2015.

Variables	Underweight—RRR (95%)	Overweight—RRR (95%)	Obese—RRR (95%)
**Intimate Partner Violence, by type**			
Physical			
Never (ref)	1	1	1
Ever	1.31 (0.66–2.62)	0.93 (0.65–1.32)	0.97 (0.59–1.59)
Emotional			
Never (ref)	1	1	1
Ever	1.41 (0.61–2.14)	1.32 (0.91–1.91)	2.22 (1.16–4.13)**
Sexual			
Never (ref)	1	1	1
Ever	1.04 (0.71–1.53)	1.14 (0.91–1.44)	1.29 (0.92–1.81)
Physical or Emotional			
Never (ref)	1	1	1
Ever	0.67 (0.25–1.78)	0.83 (0.48–1.42)	0.51 (0.22–1.81)
Physical and Emotional			
Never (ref)	1	1	1
Ever	1.13 (0.88–1.45)	0.93 (0.80–1.07)	0.76 (0.61–0.94)
Physical or Sexual			
Never (ref)	1	1	1
Ever	0.81 (0.37–1.76)	0.92 (0.61–1.37)	0.71 (0.40–1.25)
Physical and Sexual			
Never (ref)	1	1	1
Ever	1.03 (0.59–1.80)	0.80 (0.58–1.12)	0.44 (0.25–0.79)
Emotional or Sexual			
Never (ref)	1	1	1
Ever	0.78 (0.38–1.59)	0.75 (0.49–1.14)	0.37 (0.18–0.73)***
Emotional and Sexual			
Never (ref)	1	1	1
Ever	0.90 (0.48–1.67)	1.14 (0.85–1.54)	1.01 (0.67–1.53)
Physical or Emotional or Sexual			
Never (ref)	1	1	1
Ever	1.87 (0.64–5.43)	1.31 (0.72–2.37)	2.59 (1.05–6.39)*
All three			
Never (ref)	1	1	1
Ever	1.11 (0.44–2.82)	1.23 (0.74–2.04)	2.83 (1.28–6.25)

Notes: aOR- adjusted Odd Ratio. Model adjusted for women’s age, marital status, education, ethnicity, and parity, place of residence, employment status, wealth, smoking status, alcohol consumption, and media exposure.

Results from Table 3 showed that women’s exposure to any form of intimate partner violence was not significantly associated with the likelihood of being underweight or overweight relative to normal weight. However, women who had ever experienced at least one form of IPV (i.e., physical, emotional, or sexual) were more likely to be obese (aOR = 2.59; 95% CI = 1.05–6.39) relative to normal-weight women. Similarly, we found that women who had ever experienced all three forms of IPV more likely to be obese (aOR = 2.83; 95% CI = 1.28–6.25) relative to normal-weight women. The odds of being obese were also found to be higher among women with any prior exposure to emotional violence (aOR = 2.22; 95% CI = 1.16–4.13). Interestingly, the adjusted odds of being obese were lower among women who had ever experienced any emotional or sexual violence (aOR = 0.37; 95% CI = 0.18–0.73).

## Discussion

This is the first study to explore the association between Zimbabwean women’s exposure to IPV and nutritional status using ZDHS data collected from 2005–2015. Although prior studies in Zimbabwe have examined trends in the prevalence of overweight and obesity [36] as well as associations between demographic characteristics, socioeconomic status, and IPV against women [1], no study has investigated the complex relationship between IPV and nutritional status (i.e., underweight, overweight, and obesity) of women in the country. Moreover, the prevalence of both IPV and overweight is high in Zimbabwe [1,36,58,59], which makes the country an appropriate setting for this study.

Overall, the findings revealed that more than one-third (43%) of women reported to have ever experienced at least one form of intimate partner violence, which is higher than the global estimated prevalence of 30% [1,60]. These findings are consistent with previous studies in Zimbabwe [1,23,58,61,62] and other Sub-Saharan African countries [63,64]. Some of the risks for the high and increasing prevalence of IPV in developing countries have been attributed to cohabitation [65], rural residence [66,67], and low economic status [68–70]. Poverty on the other hand has been shown to be a determinant of IPV [71,72] as poor women tend to heavily depend on their partners [69,72,73], which may limit their bargaining powers.

Regarding the various forms of IPV, we found emotional and sexual violence to be the most popular forms of violence against women [58,62]. Sexual violence may be low due to underreporting of such abuses in Africa [67,74], stemming from traditional norms and beliefs [75].

The findings further revealed that women of reproductive age are at high risk of excess weight [35,76,77], as more than one-fifth reported being overweight and about 12% obese. Several studies have reported overweight and obesity to be on the rise in developing countries [33,35,36], and risk factors such as high economic status, urban residence [78,79], and, indeed, intimate partner violence [80,81] have been implicated.

Both intimate partner violence against women and obesity are growing health problems in low and middle-income countries (LMICs) [33–35,52,64,76,81]. Our findings showed that women who had ever experienced any form of IPV were more likely to be obese. Prior research have linked stressors including IPV with obesity [82]. It has been shown that stressful conditions may lead to the development of obesity through several mechanisms and pathways including increased hormone release [83,84], which can increase food cravings. [85,86]. In a study, Torres and Nowson (2007) found increased rate of obesity among people who face mild stressors [18]. This may be due to overeating and consumption of food that are in high calories or sugar [87,88], which may affect behavioural patterns such as sleep and physical activity [89]. There is some evidence that obesity affects women’s participation in daily routines [90–92] which can affect their participation in the labour market [86], and also impact other health outcomes [85,93].

Surprisingly, we did not find any significant association between IPV and underweight, relative to normal weight. While this finding is consistent with some studies [77,94], others suggest that exposure to IPV increases the odds of being underweight [94,95]. These inconsistent findings may be attributed to study population, demographic and socioeconomic contexts [18,30,94]. Meanwhile, the positive association between IPV and underweight has been associated with dietary behaviours characterized by substance abuse, insufficient calorie intake, or reduced food intake [30]. Furthermore, abusive partners may withhold food from victims, as a form of punishment that can negatively affect their weight [18,30]. These inconsistent findings call for future research to explore this issue closely.

IPV and poor nutrition (underweight and overweight) are major determinants of health [96,97], especially among women of reproductive age [98,99]. While obesity is a risk factor for non-communicable diseases such as diabetes and hypertension [100–102], IPV has been linked with mental health problems including traumatic stress [15,103,104] and injury [5,24,105]. These findings, including the results presented in the current study, should be taken into account for the development of policies aiming for the promotion of peace and security of women. Such policies need to address gender-related health issues as well as opportunities and pathways to reduce gender inequity and gendered social and health problems including IPV.

### Strengths and limitations

The major strength of this study was that a nationally representative sample was used, where participants were sampled using probability sampling methods [23]. The range of relevant questions in the survey allowed for a detailed assessment of the IPV-obesity link in a large sample of women from Zimbabwe. Nonetheless, there are some limitations. First, due to the cross-sectional design of the DHS data, causality of associations between variables cannot be established. Longitudinal studies on exposure to IPV and the association with adverse health outcomes would be better suited for causal interpretation, although the currently available survey data already provide some convincing insights into the problem under investigation. Second, it has been shown that exposure to violence during childhood may increase subsequent exposures in adulthood [80,106,107], which may lead to excess weight. However, the study lacks data on violence experienced during childhood. Third, this study used secondary data, hence, information on other imperative behavioural factors such as nutritional history and physical inactivity that might have explained the prevalence of excess weight in the study sample was not available. Fourth, DHS measures self-reported IPV, and this may under estimate IPV among participants in our sample. Finally, it is likely that IPV reporting is hampered by issues of privacy, shame, etc. This can lead to information bias, hence additional approaches to validate and enhance information on IPV experiences need to be considered [108–110].

## Conclusion

The study findings show that women of reproductive age in Zimbabwe are at high risk of both IPV and excess weight. Moreover, we found a positive relationship between exposure to at least one form of IPV and obesity. Public health interventions that target the well-being, empowerment and development of women are needed to address the complex issue of IPV and adverse health outcomes, including obesity. Legal, social and health institutions should collaborate to develop and implement appropriate intervention measures.

## References

[1] Iman’ishimwe Mukamana J, Machakanja P, Adjei NK. Trends in prevalence and correlates of intimate partner violence against women in Zimbabwe, 2005–2015. BMC International Health and Human Rights. 2020;20: 2. doi: 10.1186/s12914-019-0220-8 31959182 PMC6971918

[2] Garcia-Moreno C, Jansen HA, Ellsberg M, Heise L, Watts CH. Prevalence of intimate partner violence: findings from the WHO multi-country study on women’s health and domestic violence. The Lancet. 2006;368: 1260–1269. doi: 10.1016/S0140-6736(06)69523-8 17027732

[3] Uthman OA, Lawoko S, Moradi T. Factors associated with attitudes towards intimate partner violence against women: a comparative analysis of 17 sub-Saharan countries. BMC International Health and Human Rights. 2009;9: 14. doi: 10.1186/1472-698X-9-14 19619299 PMC2718859

[4] Goodman LA, Koss MP, Felipe Russo N. Violence against women: Physical and mental health effects. Part I: Research findings. Applied and Preventive Psychology. 1993;2: 79–89. doi: 10.1016/s0962-1849(05)80114-3

[5] Black MC. Intimate Partner Violence and Adverse Health Consequences: Implications for Clinicians. American Journal of Lifestyle Medicine. 2011;5: 428–439. doi: 10.1177/1559827611410265

[6] The Five Types of Intimate Partner Violence. In: Elite Learning [Internet]. 1 Dec 2014 [cited 6 Nov 2019]. https://www.elitecme.com/resource-center/nursing/five-types-intimate-partner-violence/.

[7] Krug EG, Mercy JA, Dahlberg LL, Zwi AB. The world report on violence and health. The Lancet. 2002;360: 1083–1088. doi: 10.1016/S0140-6736(02)11133-0 12384003

[8] Joel Yager MD. Intimate Partner Violence Can Take Many Forms. NEJM Journal Watch. 2018;2018. doi: 10.1056/nejm-jw.NA46613

[9] pubmed dev AZ et al. Income, Gender, and Forms of Intimate Partner Violence.—PubMed—NCBI. [cited 6 Nov 2019]. https://www.ncbi.nlm.nih.gov/pubmed/29294851.

[10] García-Moreno C, Jansen H a. FM, Ellsberg M, Heise L, Watts C. WHO multi-country study on women’s health and domestic violence against women: initial results on prevalence, health outcomes and women’s responses. WHO multi-country study on women’s health and domestic violence against women: initial results on prevalence, health outcomes and women’s responses. 2005 [cited 26 Sep 2018]. https://www.cabdirect.org/cabdirect/abstract/20063002089.

[11] Saunders DG. Are Physical Assaults by Wives and Girlfriends a Major Social Problem?: A Review of the Literature. Violence Against Women. 2002;8: 1424–1448. doi: 10.1177/107780102237964

[12] Bott S, Morrison A, Ellsberg M. Preventing and responding to gender-based violence in middle and low-income countries: a global review and analysis. The World Bank; 2005 Jun p. 1. Report No.: WPS3618. http://documents.worldbank.org/curated/en/852691468140377416/Preventing-and-responding-to-gender-based-violence-in-middle-and-low-income-countries-a-global-review-and-analysis.

[13] What Works to Prevent Partner Violence? An Evidence Overview | STRIVE. [cited 6 Dec 2019]. http://strive.lshtm.ac.uk/resources/what-works-prevent-partner-violence-evidence-overview.

[14] Bott S, Guedes A, Ruiz-Celis AP, Mendoza JA. Intimate partner violence in the Americas: a systematic review and reanalysis of national prevalence estimates. Rev Panam Salud Publica. 2019;43. doi: 10.26633/RPSP.2019.26 31093250 PMC6425989

[15] Coker AL, Smith PH, Bethea L, King MR, McKeown RE. Physical health consequences of physical and psychological intimate partner violence. Arch Fam Med. 2000;9: 451–457. doi: 10.1001/archfami.9.5.451 10810951

[16] Åsling-Monemi K, Peña R, Ellsberg MC, Persson LÅ. Violence against women increases the risk of infant and child mortality: a case-referent study in Nicaragua. Bull World Health Organ. 2003;81: 10–16. doi: 10.1590/S0042-96862003000100004 12640470 PMC2572309

[17] de Ferreira M F, de Moraes CL, Reichenheim ME, Verly E Junior, Marques ES, Salles-Costa R. Effect of physical intimate partner violence on body mass index in low-income adult women. Cad Saúde Pública. 2015;31: 161–172. doi: 10.1590/0102-311x00192113 25715300

[18] Yount KM, Li L. Domestic Violence and Obesity in Egyptian Women. Journal of Biosocial Science. 2011;43: 85–99. doi: 10.1017/S0021932010000441 20809993

[19] Profiling Domestic Violence; A Multi-Country Study (English). [cited 28 Nov 2019]. https://www.dhsprogram.com/publications/publication-od31-other-documents.cfm.

[20] Cools S, Kotsadam A. Resources and Intimate Partner Violence in Sub-Saharan Africa. World Development. 2017;95: 211–230. doi: 10.1016/j.worlddev.2017.02.027

[21] Ahinkorah BO. Polygyny and intimate partner violence in sub-Saharan Africa: Evidence from 16 cross-sectional demographic and health surveys. SSM Popul Health. 2021;13: 100729. doi: 10.1016/j.ssmph.2021.100729 33511263 PMC7815814

[22] Coll CVN, Ewerling F, García-Moreno C, Hellwig F, Barros AJD. Intimate partner violence in 46 low-income and middle-income countries: an appraisal of the most vulnerable groups of women using national health surveys. BMJ Global Health. 2020;5: e002208. doi: 10.1136/bmjgh-2019-002208 32133178 PMC7042580

[23] Agency ZNS, International ICF. Zimbabwe Demographic and Health Survey 2015: Final Report. 2016 [cited 16 Sep 2018]. https://dhsprogram.com/publications/publication-fr322-dhs-final-reports.cfm.

[24] Campbell J, Jones AS, Dienemann J, Kub J, Schollenberger J, O’Campo P, et al. Intimate Partner Violence and Physical Health Consequences. Arch Intern Med. 2002;162: 1157–1163. doi: 10.1001/archinte.162.10.1157 12020187

[25] Campbell JC. Health consequences of intimate partner violence. THE LANCET. 2002;359: 6. doi: 10.1016/S0140-6736(02)08336-8 11965295

[26] CAMPBELL JC, SOEKEN KL. Forced Sex and Intimate Partner Violence: Effects on Women’s Risk and Women’s Health. Violence Against Women. 1999;5: 1017–1035. doi: 10.1177/1077801299005009003

[27] Bonomi AE, Thompson RS, Anderson M, Reid RJ, Carrell D, Dimer JA, et al. Intimate Partner Violence and Women’s Physical, Mental, and Social Functioning. American Journal of Preventive Medicine. 2006;30: 458–466. doi: 10.1016/j.amepre.2006.01.015 16704938

[28] Ellsberg M, Jansen HAFM, Heise L, Watts CH, Garcia-Moreno C, WHO Multi-country Study on Women’s Health and Domestic Violence against Women Study Team. Intimate partner violence and women’s physical and mental health in the WHO multi-country study on women’s health and domestic violence: an observational study. Lancet. 2008;371: 1165–1172. doi: 10.1016/S0140-6736(08)60522-X 18395577

[29] Jewkes R, Morrell R. Gender and sexuality: emerging perspectives from the heterosexual epidemic in South Africa and implications for HIV risk and prevention. J Int AIDS Soc. 2010;13: 6. doi: 10.1186/1758-2652-13-6 20181124 PMC2828994

[30] Ackerson LK, Subramanian SV. Domestic violence and chronic malnutrition among women and children in India. Am J Epidemiol. 2008;167: 1188–1196. doi: 10.1093/aje/kwn049 18367471 PMC2789268

[31] Sethuraman K, Lansdown R, Sullivan K. Women’s Empowerment and Domestic Violence: The Role of Sociocultural Determinants in Maternal and Child Undernutrition in Tribal and Rural Communities in South India. Food Nutr Bull. 2006;27: 128–143. doi: 10.1177/156482650602700204 16786979

[32] Dutton MA, Green BL, Kaltman SI, Roesch DM, Zeffiro TA, Krause ED. Intimate Partner Violence, PTSD, and Adverse Health Outcomes. Journal of Interpersonal Violence. 2006;21: 955–968. doi: 10.1177/0886260506289178 16731994

[33] Kan K, Tsai W-D. Obesity and risk knowledge. Journal of Health Economics. 2004;23: 907–934. doi: 10.1016/j.jhealeco.2003.12.006 15353186

[34] Friedrich MJ. Global Obesity Epidemic Worsening. JAMA. 2017;318: 603–603. doi: 10.1001/jama.2017.10693 28810033

[35] Abubakari AR, Lauder W, Agyemang C, Jones M, Kirk A, Bhopal RS. Prevalence and time trends in obesity among adult West African populations: a meta-analysis. Obes Rev. 2008;9: 297–311. doi: 10.1111/j.1467-789X.2007.00462.x 18179616

[36] Mukora-Mutseyekwa F, Zeeb H, Nengomasha L, Adjei N. Trends in prevalence & determinants of overweight & obesity among women of reproductive age in Zimbabwe, 2005–2015. 2019. 10.3390/ijerph16152758 PMC6695964 31382360

[37] Lincoln KD, Abdou CM, Lloyd D. Race and Socioeconomic Differences in Obesity and Depression among Black and Non-Hispanic White Americans. J Health Care Poor Underserved. 2014;25: 257–275. doi: 10.1353/hpu.2014.0038 24509025 PMC4830390

[38] Seematter G, Dirlewanger M, Rey V, Schneiter P, Tappy L. Metabolic effects of mental stress during over- and underfeeding in healthy women. Obes Res. 2002;10: 49–55. doi: 10.1038/oby.2002.7 11786601

[39] Huang HY, Yang W, Omaye ST. Intimate partner violence, depression and overweight/obesity. Aggression and Violent Behavior. 2011;16: 108–114. doi: 10.1016/j.avb.2010.12.005

[40] Alvarez J, Pavao J, Baumrind N, Kimerling R. The Relationship Between Child Abuse and Adult Obesity Among California Women. American Journal of Preventive Medicine. 2007;33: 28–33. doi: 10.1016/j.amepre.2007.02.036 17572308

[41] Johnson PJ, Hellerstedt WL, Pirie PL. Abuse history and nonoptimal prenatal weight gain. Public Health Rep. 2002;117: 148–156. doi: 10.1093/phr/117.2.148 12356999 PMC1497424

[42] Boy A, Salihu HM. Intimate partner violence and birth outcomes: a systematic review. Int J Fertil Womens Med. 2004;49: 159–164. 15481481

[43] Irie M, Asami S, Nagata S, Miyata M, Kasai H. Relationships between perceived workload, stress and oxidative DNA damage. Int Arch Occup Environ Health. 2001;74: 153–157. doi: 10.1007/s004200000209 11317710

[44] Hapuarachchi JR, Chalmers AH, Winefield AH, Blake-Mortimer JS. Changes in clinically relevant metabolites with psychological stress parameters. Behav Med. 2003;29: 52–59. doi: 10.1080/08964280309596057 15147103

[45] Epel ES, Blackburn EH, Lin J, Dhabhar FS, Adler NE, Morrow JD, et al. From the Cover: Accelerated telomere shortening in response to life stress. Proceedings of the National Academy of Sciences of the United States of America. 2004;101: 17312. doi: 10.1073/pnas.0407162101 15574496 PMC534658

[46] Raj A, Livramento KN, Santana MC, Gupta J, Silverman JG. Victims of intimate partner violence more likely to report abuse from in-laws. Violence Against Women. 2006;12: 936–949. doi: 10.1177/1077801206292935 16957174

[47] Stephenson R, Koenig MA, Ahmed S. Domestic violence and symptoms of gynecologic morbidity among women in North India. Int Fam Plan Perspect. 2006;32: 201–208. doi: 10.1363/3220106 17237017

[48] WHO | Global status report on noncommunicable diseases 2014. In: WHO [Internet]. [cited 21 Oct 2018]. http://www.who.int/nmh/publications/ncd-status-report-2014/en/.

[49] Mathew AE, Marsh B, Smith LS, Houry D. Association between Intimate Partner Violence and Health Behaviors of Female Emergency Department Patients. Western Journal of Emergency Medicine. 2012;13: 278. doi: 10.5811/westjem.2012.3.11747 22900126 PMC3415833

[50] Mufunda E, Makuyana L. Mufunda E. and Makuyana L. (2016) Obesity: a potential pandemic among the youths in zimbabwe. Journal of Diabetes Mellitus, 6, 136–145. Journal of Diabetes Mellitus, 6, 136–145. 2016;6: 136–145. doi: 10.4236/jdm.2016.62014

[51] Barao K, Forones NM. Body mass index: different nutritional status according to WHO, OPAS and Lipschitz classifications in gastrointestinal cancer patients. Arquivos de Gastroenterologia. 2012;49: 169–171. doi: 10.1590/s0004-28032012000200013 22767006

[52] Obesity: preventing and managing the global epidemic: report of a WHO consultation. [cited 21 Oct 2018]. http://apps.who.int/iris/handle/10665/42330. 11234459

[53] Obesity. [cited 3 Jul 2020]. https://www.who.int/westernpacific/health-topics/obesity.

[54] WHO | Obesity: preventing and managing the global epidemic. In: WHO [Internet]. World Health Organization; [cited 9 Jun 2020]. http://www.who.int/entity/nutrition/publications/obesity/WHO_TRS_894/en/index.html.

[55] Chapman H, Gillespie SM. The Revised Conflict Tactics Scales (CTS2): A review of the properties, reliability, and validity of the CTS2 as a measure of partner abuse in community and clinical samples. Aggression and Violent Behavior. 2019;44: 27–35. doi: 10.1016/j.avb.2018.10.006

[56] Filson J, Ulloa E, Runfola C, Hokoda A. Does Powerlessness Explain the Relationship Between Intimate Partner Violence and Depression? J Interpers Violence. 2010;25: 400–415. doi: 10.1177/0886260509334401 19487687

[57] WHO | Putting women first: Ethical and safety recommendations for research on domestic violence against women. [cited 23 Oct 2018]. https://www.who.int/gender-equity-rights/knowledge/who_fch_gwh_01.1/en/.

[58] Shamu S, Zarowsky C, Roelens K, Temmerman M, Abrahams N. High-frequency intimate partner violence during pregnancy, postnatal depression and suicidal tendencies in Harare, Zimbabwe. General Hospital Psychiatry. 2016;38: 109–114. doi: 10.1016/j.genhosppsych.2015.10.005 26607330

[59] Biadgilign S, Mgutshini T, Haile D, Gebremichael B, Moges Y, Tilahun K. Epidemiology of obesity and overweight in sub-Saharan Africa: a protocol for a systematic review and meta-analysis. BMJ Open. 2017;7: e017666. doi: 10.1136/bmjopen-2017-017666 29175884 PMC5719333

[60] WHO | Global and regional estimates of violence against women. In: WHO [Internet]. [cited 15 Sep 2018]. http://www.who.int/reproductivehealth/publications/violence/9789241564625/en/.

[61] Fidan A, Bui HN. Intimate Partner Violence Against Women in Zimbabwe: Violence Against Women. 2015 [cited 8 Jul 2020]. doi: 10.1177/1077801215617551 26644331

[62] Ezechi OC, Kalu BK, Ezechi LO, Nwokoro CA, Ndububa VI, Okeke GCE. Prevalence and pattern of domestic violence against pregnant Nigerian women. Journal of Obstetrics and Gynaecology. 2004;24: 652–656. doi: 10.1080/01443610400007901 16147605

[63] Global and regional estimates of violence against women: prevalence and health effects of intimate partner violence and non-partner sexual violence. Geneva: World Health Organization, Department of Reproductive Health and Research; 2013.

[64] Garcia-Moreno C, Jansen HAFM, Ellsberg M, Heise L, Watts CH, WHO Multi-country Study on Women’s Health and Domestic Violence against Women Study Team. Prevalence of intimate partner violence: findings from the WHO multi-country study on women’s health and domestic violence. Lancet. 2006;368: 1260–1269. doi: 10.1016/S0140-6736(06)69523-8 17027732

[65] Jackson NA. Observational experiences of intrapersonal conflict and teenage victimization: A comparative study among spouses and cohabitors. Journal of Family Violence. 1996;11: 191–203. doi: 10.1007/BF02336940

[66] Ajah LO, Iyoke CA, Nkwo PO, Nwakoby B, Ezeonu P. Comparison of domestic violence against women in urban versus rural areas of southeast Nigeria. International Journal of Women’s Health. 2014;6: 865. doi: 10.2147/IJWH.S70706 25336992 PMC4199982

[67] Hindin MJ. Understanding women’s attitudes towards wife beating in Zimbabwe. Bull World Health Organ, Bull World Health Organ. 2003;81: 501–508. doi: 10.1590/S0042-96862003000700008 12973642 PMC2572507

[68] Hornung CA, McCullough BC, Sugimoto T. Status Relationships in Marriage: Risk Factors in Spouse Abuse. Journal of Marriage and Family. 1981;43: 675–692. doi: 10.2307/351768

[69] Cunradi CB, Caetano R, Clark C, Schafer J. Neighborhood Poverty as a Predictor of Intimate Partner Violence Among White, Black, and Hispanic Couples in the United States: A Multilevel Analysis. Annals of Epidemiology. 2000;10: 297–308. doi: 10.1016/s1047-2797(00)00052-1 10942878

[70] Lawoko S, Dalal K, Jiayou L, Jansson B. Social Inequalities in Intimate Partner Violence: A Study of Women in Kenya. Violence and Victims. 2007;22: 773–784. doi: 10.1891/088667007782793101 18225388

[71] YLLÖ K. Sexual Equality and Violence Against Wives in American States. Journal of Comparative Family Studies. 1983;14: 67–86. Available: https://www.jstor.org/stable/41601328.

[72] Heise LL. Determinants of partner violence in low and middle-income countries: exploring variation in individual and population-level risk. doctoral, London School of Hygiene & Tropical Medicine. 2012. http://researchonline.lshtm.ac.uk/682451/.

[73] Vyas S, Watts C. How does economic empowerment affect women’s risk of intimate partner violence in low and middle income countries? A systematic review of published evidence. Journal of International Development. 2009;21: 577–602. doi: 10.1002/jid.1500

[74] Dobash RE, Dobash RP. Wives: The appropriate victims of marital violence. Victimology. 1977;2: 426–442.

[75] Jewkes R, Morrell R. Sexuality and the limits of agency among South African teenage women: theorising femininities and their connections to HIV risk practices. Soc Sci Med. 2012;74: 1729–1737. doi: 10.1016/j.socscimed.2011.05.020 21696874 PMC3217103

[76] Mitchell N, Catenacci V, Wyatt HR, Hill JO. OBESITY: OVERVIEW OF AN EPIDEMIC. Psychiatr Clin North Am. 2011;34: 717–732. doi: 10.1016/j.psc.2011.08.005 22098799 PMC3228640

[77] Davies R, Lehman E, Perry A, McCall-Hosenfeld JS. Association of intimate partner violence and health-care provider-identified obesity. Women & Health. 2016;56: 561–575. doi: 10.1080/03630242.2015.1101741 26495745 PMC5808410

[78] Herald T. Is Zimbabwe sliding towards obesity? In: The Herald [Internet]. [cited 7 Nov 2019]. https://www.herald.co.zw/is-zimbabwe-sliding-towards-obesity/.

[79] Chronicle T. A fat nation: Urban Zimbabwe’s descent to obesity. In: The Chronicle [Internet]. [cited 7 Nov 2019]. https://www.chronicle.co.zw/a-fat-nation-urban-zimbabwes-descent-to-obesity/.

[80] Midei AJ, Matthews KA. Interpersonal violence in childhood as a risk factor for obesity: a systematic review of the literature and proposed pathways. Obes Rev. 2011;12: e159–172. doi: 10.1111/j.1467-789X.2010.00823.x 21401850 PMC3104728

[81] Bosch J, Weaver TL, Arnold LD, Clark EM. The Impact of Intimate Partner Violence on Women’s Physical Health: Findings From the Missouri Behavioral Risk Factor Surveillance System. J Interpers Violence. 2017;32: 3402–3419. doi: 10.1177/0886260515599162 26268271

[82] Dallman MF. Stress-induced obesity and the emotional nervous system. Trends Endocrinol Metab. 2010;21: 159–165. doi: 10.1016/j.tem.2009.10.004 19926299 PMC2831158

[83] Boeckel MG, Viola TW, Daruy-Filho L, Martinez M, Grassi-Oliveira R. Intimate partner violence is associated with increased maternal hair cortisol in mother-child dyads. Compr Psychiatry. 2017;72: 18–24. doi: 10.1016/j.comppsych.2016.09.006 27693887

[84] van der Meij L, Pulopulos MM, Hidalgo V, Almela M, Lila M, Roney JR, et al. Hormonal changes of intimate partner violence perpetrators in response to brief social contact with women. Aggressive Behavior. 2022;48: 30–39. doi: 10.1002/ab.21995 34605041 PMC9293448

[85] Gordon‐Larsen P. Obesity-Related Knowledge, Attitudes, and Behaviors in Obese and Non-obese Urban Philadelphia Female Adolescents. Obesity Research. 2001;9: 112–118. doi: 10.1038/oby.2001.14 11316345

[86] DiBonaventura M, Lay AL, Kumar M, Hammer M, Wolden ML. The Association Between Body Mass Index and Health and Economic Outcomes in the United States. J Occup Environ Med. 2015;57: 1047–1054. doi: 10.1097/JOM.0000000000000539 26461859

[87] Morales ME, Berkowitz SA. The Relationship between Food Insecurity, Dietary Patterns, and Obesity. Curr Nutr Rep. 2016;5: 54–60. doi: 10.1007/s13668-016-0153-y 29955440 PMC6019322

[88] Mason SM, Wright RJ, Hibert EN, Spiegelman D, Jun H-J, Hu FB, et al. Intimate Partner Violence and Incidence of Type 2 Diabetes in Women. Diabetes Care. 2013;36: 1159–1165. doi: 10.2337/dc12-1082 23248189 PMC3631851

[89] Tomiyama AJ. Stress and Obesity. 2018; 16. 10.1146/annurev-psych-010418-102936 29927688

[90] Pudrovska T, Reither EN, Logan ES, Sherman-Wilkins KJ. Gender and reinforcing associations between socioeconomic disadvantage and body mass over the life course. J Health Soc Behav. 2014;55: 283–301. doi: 10.1177/0022146514544525 25138198 PMC4198174

[91] Conklin AI, Forouhi NG, Suhrcke M, Surtees P, Wareham NJ, Monsivais P. Socioeconomic status, financial hardship and measured obesity in older adults: a cross-sectional study of the EPIC-Norfolk cohort. BMC Public Health. 2013;13: 1039. doi: 10.1186/1471-2458-13-1039 24188462 PMC4228357

[92] Hiilamo A, Lallukka T, Mänty M, Kouvonen A. Obesity and socioeconomic disadvantage in midlife female public sector employees: a cohort study. BMC Public Health. 2017;17: 842. doi: 10.1186/s12889-017-4865-8 29065863 PMC5655943

[93] Djalalinia S, Qorbani M, Peykari N, Kelishadi R. Health impacts of Obesity. Pak J Med Sci. 2015;31: 239–242. doi: 10.12669/pjms.311.7033 25878654 PMC4386197

[94] Ferdos J, Rahman M, Ferdos J, Rahman M. Exposure to intimate partner violence and malnutrition among young adult Bangladeshi women: cross-sectional study of a nationally representative sample. Cadernos de Saúde Pública. 2018;34. doi: 10.1590/0102-311X00113916 30088578

[95] Sivonová M, Zitnanová I, Hlincíková L, Skodácek I, Trebatická J, Duracková Z. Oxidative stress in university students during examinations. Stress. 2004;7: 183–188. doi: 10.1080/10253890400012685 15764015

[96] Adhikari RP, Yogi S, Acharya A, Cunningham K. Intimate partner violence and nutritional status among nepalese women: an investigation of associations. BMC Womens Health. 2020;20: 127. doi: 10.1186/s12905-020-00991-x 32552716 PMC7301521

[97] Rahman M, Nakamura K, Seino K, Kizuki M. Intimate partner violence and chronic undernutrition among married Bangladeshi women of reproductive age: are the poor uniquely disadvantaged? Eur J Clin Nutr. 2013;67: 301–307. doi: 10.1038/ejcn.2012.202 23232590

[98] Willie TC, Kershaw TS, Callands TA. Examining relationships of intimate partner violence and food insecurity with HIV-related risk factors among young pregnant Liberian women. AIDS Care. 2018;30: 1156–1160. doi: 10.1080/09540121.2018.1466983 29682990 PMC6037546

[99] Diamond-Smith N, Conroy AA, Tsai AC, Nekkanti M, Weiser SD. Food insecurity and intimate partner violence among married women in Nepal. J Glob Health. 2019;9: 010412. doi: 10.7189/jogh.09.010412 30774941 PMC6359930

[100] Oğuz A, Temizhan A, Abaci A, Kozan O, Erol C, Ongen Z, et al. Obesity and abdominal obesity; an alarming challenge for cardio-metabolic risk in Turkish adults. Anadolu Kardiyol Derg. 2008;8: 401–406. 19103534

[101] Pi-Sunyer X. The Medical Risks of Obesity. Postgrad Med. 2009;121: 21–33. doi: 10.3810/pgm.2009.11.2074 19940414 PMC2879283

[102] Rubenstein AH. Obesity: a modern epidemic. Trans Am Clin Climatol Assoc. 2005;116: 103–111; discussion 112–113. 16555609 PMC1473136

[103] Tjaden P, Thoennes N. Prevalence and consequences of male-to-female and female-to-male intimate partner violence as measured by the National Violence Against Women Survey. Violence Against Women. 2000;6: 142–161. doi: 10.1177/10778010022181769

[104] Goodman LA, Koss MP, Felipe Russo N. Violence against women: Physical and mental health effects. Part I: Research findings. Applied and Preventive Psychology. 1993;2: 79–89. doi: 10.1016/S0962-1849(05)80114-3

[105] Breiding MJ, Black MC, Ryan GW. Chronic Disease and Health Risk Behaviors Associated with Intimate Partner Violence—18 U.S. States/Territories, 2005. Annals of Epidemiology. 2008;18: 538–544. doi: 10.1016/j.annepidem.2008.02.005 18495490

[106] Alhalal E. Obesity in women who have experienced intimate partner violence. Journal of Advanced Nursing. 2018;74: 2785–2797. doi: 10.1111/jan.13797 30019424

[107] Whitaker RC, Phillips SM, Orzol SM, Burdette HL. The association between maltreatment and obesity among preschool children. Child Abuse & Neglect. 2007;31: 1187–1199. doi: 10.1016/j.chiabu.2007.04.008 18023869 PMC2621258

[108] Testa M, Livingston JA, VanZile-Tamsen C. ADVANCING THE STUDY OF VIOLENCE AGAINST WOMEN USING MIXED METHODS: INTEGRATING QUALITATIVE METHODS INTO A QUANTITATIVE RESEARCH PROGRAM. Violence Against Women. 2011;17: 236–250. doi: 10.1177/1077801210397744 21307032 PMC3053530

[109] Matthews S. Crafting Qualitative Research Articles on Marriages and Families. Journal of Marriage and the Family. 2005;67: 799–808. doi: 10.1111/j.1741-3737.2005.00176.x

[110] Ruiz‐Pérez I, Plazaola‐Castaño J, Vives‐Cases C. Methodological issues in the study of violence against women. J Epidemiol Community Health. 2007;61: ii26–ii31. doi: 10.1136/jech.2007.059907 18000113 PMC2465770

