# Supplementary material for: Investigating the associations between intimate partner violence and nutritional status of women in Zimbabwe
Source: PLoS One. 2022 Jul 25;17(7):e0272038. doi: 10.1371/journal.pone.0272038 (PMC9312962; doi:10.1371/journal.pone.0272038)
Supplement: Attachment — Submitted filename: Response to Reviewers.docx [file pone.0272038.s001.docx]

**Manuscript reference number: PONE-D-21-11105**

**Title: Investigating the associations between intimate partner violence and nutritional status of women in Zimbabwe**

Dear Editor,

Thank you for the feedback on our manuscript and for the opportunity to respond to the reviewers’ comments.

We were encouraged that the reviewers felt that the results were important contribution to the literature. We have responded in detail to each of the helpful reviewer comments below.

In summary the reviewers suggest that the introduction should outline the state of the art regarding studies on IPV and nutritional status. They also requested some further clarification around our study variables. Their suggestions are helpful and we have addressed the issues in full in the revised draft.

Thank you and the reviewers for your time and consideration in assisting us to improve this manuscript. We feel that our paper will be of great relevance to *Plos One* and the readers and we look forward to hearing from you.

Kind regards,

Nicholas Kofi Adjei, PhD (*on behalf of all authors*)

1. **Reviewer #1: Overall comments**

The thematic of the manuscript is interesting and relevant, given the scarcity of studies on the relationship between IPV and inadequate nutritional status.

Response: *Thank you.*

1.1 **Major Comments: Abstract**

**1.1a**. I suggest revising the wording of the passage “Relative to normal weight, women who had ever experienced at least one form of IPV (i.e., physical, emotional, or sexual) were more likely to be obese (aOR = 2.59; 95% CI = 1.05–6.39). Women’s exposure to any form of intimate partner violence was not significantly associated with the likelihood of being underweight or overweight relative to normal weight.”, because it is confusing since, in the conclusion of the abstract, you state that “we found a positive relationship between IPV and obesity.”

**Response:** *Thank you for this comment, we have now revised the wording in the abstract.*

*Page 1, line39-43: “Relative to normal weight, women who had ever experienced at least one form of IPV (i.e., physical, emotional, or sexual) were more likely to be obese (aOR = 2.59; 95% CI = 1.05–6.39). Women’s exposure to any form of intimate partner violence was not significantly associated with the likelihood of being underweight or overweight relative to normal weight.” And Line 45-46: “between exposure to at least one form IPV and obesity.”*

**1.2. Comment: Introduction**

**1.2a.**The introduction lacks information about the state of the art regarding studies on intimate partner violence and nutritional status in order to point out what has been studied and the gaps.

**Response:** *Thank you. We have added more literature to point out what has been studied and the existing gaps regarding research on intimate partner violence and nutritional status.*

*Page 2, line 68-73: “The prevalence of IPV is high in developing countries [1]. However, there is evidence of cross-country variations [20,21], where Zimbabwe has been found to be one of the countries in sub-Saharan Africa with the highest prevalence of IPV [21,22]. It is estimated that approximately 35% of women had experienced physical violence from the age of 15 and 14% had experienced sexual violence [23]. In a recent study in Zimbabwe, Mukamana and colleagues found a substantial rise in the prevalence of IPV from 40.9% in 2010 to 43.1% in 2015 [1].”*

*Line 83-86: “In Zimbabwe, for instance, a recent study showed an increase in the prevalence of overweight and obesity from 25.0% in 2005 to 36.6% in 2015 [36]. The authors also observed socioeconomic and demographic differences in overweight and obesity among women of reproductive age.”*

**1.3. Comment: Methods**

**1.3ai.**The authors could make it clear whether the full Revised Conflict Tactics (CTs) instrument or single questions were used for the IPV measurement. If the authors used single questions, this should be included as one of the limitations of the paper and how this form of data collection impacts the findings.

**Response:** *Thank you, the Modified Conflict Tactic scale was used for IPV measurement, in line with previous studies.*

*Page 3, line 132-135: “We used the measurement of IPV in the surveys that was based on the modified Conflict Tactics (CTS2).”*

**1.3aii.**Is the marital status only these two answer options (married, cohabiting)? And women who are dating and the partner does not reside at the household.

**Response:** *Thanks. This study considerered only those in unions (living together with their partners) – i.e., either married or cohabiting.*

**1.4. Comments: Results**

**1.4ai.** RESULT

The main finding of the paper which is the positive association of emotional IPV with obesity is "erased" by the other analyses which involve the possibility of having 1 of the two types of violence (physical OR emotional; physical OR sexual, ...). I think the analysis could be more objective and contemplate the role of each type of violence separately or their coexistence.

Response: *Thank you for your comment. We have now assessed the co-occurrence of the various forms of violence. (see table 1, 2 and 3) and the result section.*

*Page 5-6, line 217-219: “Similarly, we found that women who had ever experienced all three forms of IPV more likely to be obese (aOR = 2.83; 95% CI = 1.28–6.25) relative to normal-weight women.”*

**1.4aii**. Table 1: insert 95% confidence interval

**Response:** *We have now added 95% confidence interval (See table1)*

**1.4aiii**.Table 2: insert 95% confidence interval

**Response***: Thanks.* *We have now added 95% confidence interval (See table1)*

**1.4aiv.** In table 2 it would be more interesting to bring the specific types of violence (emotional, physical, and sexual) and the co-occurrence emotional AND physical, emotional AND sexual and this way on.

**Response*:*** *Thank you for your comment. We have now included the co-occurrences of the various forms of violence (table 2)*

**1.4av.**Table 3. It would be more interesting to bring the specific types of violence (emotional, physical, and sexual) and the co-occurrence emotional AND physical, emotional AND sexual and this way on

**Response:** *Thanks. We have now included the co-occurrences of the various forms of violence (table 3)*

**1.4avi.** Figure 1

It would be interesting to bring the proportion of physical, emotional, or sexual violence against women of reproductive age (15–49 years) by nutritional status the year 2005/2006, 2010/2011, and 2015

**Response:** *Thank you. We have now included the proportion of physical, emotional, or sexual violence against women of reproductive age (15–49 years) by nutritional status the year 2005/2006, 2010/2011, and 2015 ( See figure 2).*

**1.5 Comments: Discussion**

**1.5ai.**The discussion could delve into the possible mechanisms that may explain the relationship found (IPV positively associated with obesity)

.

**Response:** *Thanks for this comment: we have now added more possible mechanisms to explain the positive association of IPV with obesity.*

*Page 6, line 252-259: “Prior research have linked stressors including IPV with obesity [82]. It has been shown that stressful conditions may lead to the development of obesity through several mechanisms and pathways including increased hormone release [83,84], which can increase food cravings. [85,86]. In a study, Torres and Nowson (2007) found increased rate of obesity among people who face mild stressors [18]. This may be due to overeating and consumption of food that are in high calories or sugar [87,88], which may affect behavioural patterns such as sleep and physical activity [89].”*

**1.5aii.** Insert as a limitation how to measure the exposure of interest (IPV).

**Response:** *Thank you. We have included this suggestion in the limitation section.*

*Page 7, line 293-294: “Fourth, DHS measures self-reported IPV, and this may under*

*estimate IPV among participants in our sample”*

**Reviewer #2: Major Comments**

1.1. **Comment: Introduction**

**1.1a.**The introduction is short and I think this is a good option. However, I suggest contextualizing better the IPV situation in Zimbabwe.

**Response:** *Thank you for your comment. We have revised the introduction to contextualize the IPV situation in Zimbabwe and added more literature to contextualize this issue in Zimbabwe.*

*Page 2, line 68-73: “The prevalence of IPV is high in developing countries [1]. However, there is evidence of cross-country variations [20,21], where Zimbabwe has been found to be one of the countries in sub-Saharan Africa with the highest prevalence of IPV [21,22]. It is estimated that approximately 35% of women had experienced physical violence from the age of 15 and 14% had experienced sexual violence [23]. In a recent study in Zimbabwe, Mukamana and colleagues found a substantial rise in the prevalence of IPV from 40.9% in 2010 to 43.1% in 2015 [1].”*

**1.2 Comment: Method**

**1.2a.**The covariates measurement could be described in the method.

**Response:** *Thank you. We have now described the computed measures.*

*Page 4, line 153-155. “The wealth index in the DHS is usually computed using durable goods, household characteristics and basic services”*

**1.2b.**A theoretical model (DAG) could be shown in the methods section

**Response:** *We have now included DAG in the method section (See figure 1)*

**1.2c**.Table 1 – Table 1 could inform the number of women in each category, and the confidence interval.

**Response:** *Thank you, we have included the number of women in each category, and the confidence interval (see table 1)*

**1.2d**.Table 2 – table 2 could present the prevalence by BMI status

**Response***: Thank you for your comment. Table 2 is presented by BMI status (i.e., over weight and obesity vs the other groups). We feel that dichotomising it is easier for interpretation.*
